# Phosphorylation of SET Protein at Ser171 by Protein Kinase D2 Diminishes Its Inhibitory Effect on Protein Phosphatase 2A

**DOI:** 10.1371/journal.pone.0051242

**Published:** 2012-12-14

**Authors:** Atsushi Irie, Kumiko Harada, Norie Araki, Yasuharu Nishimura

**Affiliations:** 1 Department of Immunogenetics, Graduate School of Medical Sciences, Kumamoto University, Honjo, Kumamoto, Japan; 2 Department of Tumor Genetics and Biology, Graduate School of Medical Sciences, Kumamoto University, Honjo, Kumamoto, Japan; Johns Hopkins School of Medicine, United States of America

## Abstract

We previously reported that protein kinase D2 (PKD2) in T cells is promptly activated after T-cell receptor (TCR) stimulation and involved in the activation of interleukin-2 promoter and T cell death, and that one of its candidate substrate is SET protein, a natural inhibitor for protein phosphatase 2A (PP2A). In this study, we investigated the target amino acid residues of SET phosphorylated by PKD2 and the effects of phosphorylation of SET on PP2A phosphatase activity. *In vitro* kinase assay using various recombinant SET mutants having Ser/Thr to Ala substitutions revealed that Ser171 of SET is one of the sites phosphorylated by PKD2. Recombinant SET with phosphorylation-mimic Ser171 to Glu substitution reduced its inhibitory effects on PP2A phosphatase activity compared with Ser171 to Ala substituted or wild-type SET. In addition, knockdown of PKD2 in Jurkat cells by RNAi or treatment of human CD4^+^ T cell clone with the PKD2 inhibitor Gö6976 resulted in reduced PP2A activity after TCR-stimulation judged from phosphorylation status of Tyr307 of the catalytic subunit of PP2A. These results suggest that PKD2 is involved in the regulation of PP2A activity in activated T cells through phosphorylation of Ser171 of SET.

## Introduction

The phosphoprotein SET (also known as I_2_^PP2A^, IGAAD, and TAF-1β) is localized in the nucleus and cytoplasm and has a critical role in the regulation of normal and cancerous signal transduction. For example, SET protein together with another nuclear phosphoprotein pp32 forms a subunit of inhibitor for histone acetyltransferase complex with “histone masking” activity, and binding of these proteins to histones prevents their acetylation by transcriptional coactivators [1]. SET is also known as a potent inhibitor of protein phosphatase 2A (PP2A) activity [2], even though SET has also been described as an inhibitor of the tumor suppressor NM23-H1 that is a granzyme A DNase-activated factor [3]. PP2A is the major cellular serine/threonine phosphatase involved in the regulation of a variety of cellular processes and signal transduction pathways [4], [5], yet the regulation of inhibitory function of SET on PP2A activity remains to be explored.

Protein kinase D2 (PKD2) is a member of PKD family protein serine/threonine kinases and expressed in various cell types [6]. In T cells, another PKD family kinase PKD1 (also known as PKCμ) is activated upon T cell receptor (TCR)-stimulation and has been reported to be involved in thymocyte development [7], [8], [9], [10], [11]: PKD1 enhanced double-negative 3 to double-negative 4 transition of thymocytes when it localized in the plasma membrane whereas it suppressed TCRβ-rearrangement when it localized in the cytoplasm [8]. Phosphorylation of histone deacetylase (HDAC) 7 by PKD1 resulted in the activation of Nur77, which regulates thymocyte apoptosis in negative and positive selection [10], [11]. We found that PKD2 is much more abundantly present than PKD1 and PKD3 in human T cells and mouse thymocytes [12]. In human leukemic T cell line Jurkat, PKD2 is mainly present in cytoplasm and its kinase activity was up-regulated by TCR-stimulation, after which a part of PKD2 relocated into the nucleus [9]. The nuclear accumulation of activated PKD2 was also reported in human gastric cancer cells after binding of gastrin to the cholecystokinin 2 receptor, which resulted in the phosphorylation of HDAC7 [13].

Over-expression of PKD2 and TCR-stimulation up-regulated interleukin-2 promoter activity in Jurkat cells [12]. Matthews *et al.* also showed that the kinase activity of PKD2 was important for effecter cytokine production after TCR engagement [14]. In DT40 avian B cell lines, both PKD1 and PKD3 were reported to be indispensable for phosphorylation and thus for nuclear export of HDAC 5 and 7 [15], and for Hsp27 phosphorylation [16], although the PKDs were not necessary for DT40 cells to be viable and proliferate normally [16]. Recently, it was reported that a mutant of *C. elegans* lacking DKF-2, an ortholog of mammalian PKD, was more susceptible for pathogenic bacterial infection and DKF-2 was shown to be positively involved in innate immunity in *C. elegans* [17].

Whereas those observations suggest the positive role of PKDs in immune responses, it was shown that PKD2 was responsible for the interferon α-induced phosphorylation of a degron of the α interferon receptor and therefore for its accelerated degradation by ubiquitination, which could neutralize the defensive functions of type I IFN [18].

In the present work, we identified one of the phosphorylation sites of SET protein by PKD2 and potential role of SET phosphorylation by PKD2 is discussed.

## Methods

### Cells and reagents

Human leukemic T cell line Jurkat (E6-1) was obtained from American Type Culture Collection (Manassas, VA). Jurkat cells expressing GFP-tagged constitutively active (CA) mutant and GFP-tagged kinase dead (KD) mutant of PKD2 (CA-PKD2-GFP and KD-PKD2-GFP, respectively) were prepared as reported previously [12]. Jurkat cells expressing GFP-tagged wild-type SET (GFP-SET-WT) and SET S171A mutant (GFP-SET-S171A) were prepared by introducing pAcGFP1-C1 vector (Clontech, Mauntain View, CA) inserted with SETβ cDNA and the mutant SETβ cDNA, respectively. Anti-human CD3ε mAb (UCHT-1) and anti-GFP antibody were purchased from BD Biosciences (Franklin Lakes, NJ). Anti-human PKD2 antibody and anti-human SET antibody were from Bethyl Laboratories (Montgomery, TX). Anti-PP2A C-subunit phospho-Tyr307 antibody, anti-phospho-P44/42 MAPK (Thr202/Tyr204) and anti-ERK1 (K-23) sc-94 antibodies were from Epitomics (Burlingame, CA), Cell signaling (Danvers, MA) and Santa Cruz Biotechnology (Santa Cruz, CA), respectively. Anti-mouse IgG F(ab′)_2_ fragment was from PIERCE Biotechnology (Rockford, IL). Myelin basic protein (MBP, dephosphorylated), anti-PP2A C-subunit antibody and purified PP2A AC heterodimer were obtained from Upstate Biotechnology (Lake Placid, NY). Gö6976 and Gö6983 were from Calbiochem (San Diego, CA). Jurkat cell nuclear extract was purchased from Active Motif (Carlsbad CA). Poly-L-Lysine Agarose and phytohemagglutinin (PHA) were form Sigma-Aldrich (St. Louis, MO).

### Plasmid

Full-length human SETβ cDNA was obtained from total RNA of Jurkat cell using RT-PCR and inserted into a pET28a vector (Novagen, Darmstadt, Germany) for preparation of recombinant proteins or a pAcGFP1-C1 vector for Jurkat cell-transfection. Mutations of SET were introduced by overlapping PCR.

### Cell stimulation

Jurkat cells were stimulated with anti-CD3ε mAb (2 µg/ml) for 10 min followed by addition of anti-mouse IgG F(ab′)_2_ fragment (8 µg/ml) and stimulated for 1 h at 37°C, or stimulated with PHA (2 µg/ml) at 37°C for 12 hrs. Human CD4^+^ T cell clone was stimulated by co-culturing with mouse L cells expressing its cognate ligand [19] HLA-DR4 (DRB1*04:06) covalently linked with streptococcal M12p54-68 peptide for indicated periods as described previously [9], [12], [20], [21].

### Quantitaion of phosphorylated SET

Jurkat cells expressing GFP-SET-WT were stimulated by PHA and lysed using a lysis buffer supplied with a PhosphoProtein Purification Kit (Qiagen) according to the manufacturer's instruction. In some experiments, cells were treated under the presence of PKD2 inhibitor Gö6976 (3 µM) before and during the PHA stimulation or the cells were treated with siRNA as described below before the stimulation. The fluorescence of GFP in the lysate was measured at excitation 475 nm and emission 505 nm using a Hitachi Fluorescence Spectrometer F-2500 (Tokyo, Japan). Each lysate containing the same amount of fluorescence was applied to the PhosphoProtein Purification Columns (Qiagen). Phospho-GFP-SET-WT recovered was subjected to SDS-PAGE and transferred onto a PVDF membrane. The phospho-GFP-SET-WT was detected and quantified by anti-GFP antibody and horseradish peroxidase-conjugated secondary antibody followed by enhanced chemiluminescent detection (ECL, Amersham Biosciences).

### 2D-gel electrophoresis and mass-spectrometric analysis

Jurkat cells (1×10^7^) treated with anti-CD3ε mAb and anti-mouse IgG F(ab′)_2_ fragment were lysed and immunoprecipitated with anti-PKD2 antibody as described previously [12]. After washing, the beads were incubated in the kinase buffer (30 mM Tris-HCl, pH 7.4, 10 mM MgCl_2_ and 1 mM DTT) containing 16 µg of heat-inactivated (50°C, 5 min) Jurkat nuclear extract (Active Motif) and either 0.25 MBq [γ-^32^P]-ATP or 25 µM cold ATP at 30°C for 10 min. The kinase reaction with [γ-^32^P]-ATP was performed in the presence of 3 µM of Gö6983 or Gö6976. Both kinase reaction mixtures with hot and cold ATPs were submitted to isoelectric focusing on pH 3–10 non-linear immobilized pH gradient strips (7 cm, Bio-Rad) followed by 11% second dimension SDS- PAGE in parallel. After the gels were stained with syproruby reagent (Bio-Rad), the gels with radioactivity were sandwiched with clear cellophane sheets, dried and exposed to X-ray films (Fujifilm, Tokyo, Japan). UV-illuminated dried gel sheets were overlaid onto the developed X-ray films to identify radioactive protein spots. The corresponding syproruby-stained protein spots on the cold gels were excised and subjected to trypsin-digestion and mass-spectrometric analysis.

### Expression and purification of recombinant SET proteins

pET28a-SET plasmids were introduced into BL21(DE3) *E. coli* strain (Novagen). The 6×His-tagged SET protein expression was induced according to the manufacturer's instruction. The bacterial pellet was sonicated and the soluble recombinant SET proteins were fractionated by poly-L-lysine-agarose beads column according to the method described by Li *et al.* [2]. Fractions with recombinant SET proteins were combined, dialyzed and applied to a Ni-NTA resin column (0.5 ml, Qiagen). The SET protein was eluted with a 20 mM Tris-HCl (pH 8.0) buffer containing 500 mM NaCl and 500 mM imidazole. The fractions were combined and dialyzed against 50 mM Tris-HCl, pH 7.0.

### *In vitro* PP2A phosphatase assay

Dephosphorylated MBP was ^32^P-labeled by recombinant PKA (New England BioLabs) according to the manufacturer's instruction. ^32^P-MBP, recombinant SET proteins and purified PP2A (Upstate) were diluted with phosphatase buffer (50 mM Tris-HCl, pH 7.0, 50 µg/ml BSA, 1 mM DTT, 0.25 mM MnCl_2_). After mixing 16 µl of indicated concentrations of the recombinant SET and 2 µl PP2A (37 µU) and incubating at 4°C for 15 min, 2 µl ^32^P-MBP was added and left at 37°C for 10 min. The reaction was stopped by adding 6 µl of sample buffer and 20 µl of the reaction mixture was subjected to 15% SDS-PAGE. The gels were dried and the remaining radioactivity of MBP was quantified by BAS-2000 image analyzer (Fujifilm). In *ex vivo* experiments, the PP2A phosphatase activity from Jurkat or human CD4^+^ T cell clone were estimated by the phosphorylation status at Tyr307 of the catalytic subunit of PP2A by western blotting using anti-PP2A C-subunit phospho-Tyr307 [22].

### Knockdown of PKD2 by RNA interference

siRNAs for human *PKD2* were designed as follows: siRNA1 sense, 5′-GACAAACUGCGCUUCCCUACC-3′ and antisense, 5′-UAGGGAAGCGCAGUUUGUCAA-3′; siRNA2 sense, 5′- GUUCGAGACGCCUGAGAAAGU-3′ and antisense, 5′- UUUCUCAGGCGUCUCGAACAU-3′. Three µl of each duplex siRNA (100 µM) was added to Jurkat cell suspension (1.5×10^7^/100 µl) and electroporated with a Nucleofector II (Lonza) according to the manufacturer's instructions. The siRNA1 had no effect on PKD2 expression level and used as a negative control.

### Western blotting

Jurkat cells (1.5×10^6^) or human CD4^+^ T cell clone (1.5×10^6^) were lyzed with 15 µl of the lysis buffer (1% NP-40, 0.1% SDS, 0.5% sodium deoxycholate, 10% glycerol, 20 mM Tris/HCl-pH 7.4, 150 mM NaCl, 1 mM Na_3_VO_4_, 10 mM NaF, 1 mM EDTA, 1 mM EGTA, and a protease inhibitor cocktail tablet from Roche) and the supernatant was subjected to SDS-PAGE. The proteins were electro-blotted onto a PVDF membrane. PKD2, SET, GFP-tagged recombinant SET proteins, ERK1, phospho-ERK and β-actin were probed with respective antibodies and horseradish peroxidase-conjugated secondary antibody followed by the ECL detection. A lumino image analyzer (LAS-4000, Fujifilm) was used to quantify the chemiluminescence.

## Results

### SET protein is a candidate substrate for PKD2

**Figure 1:**
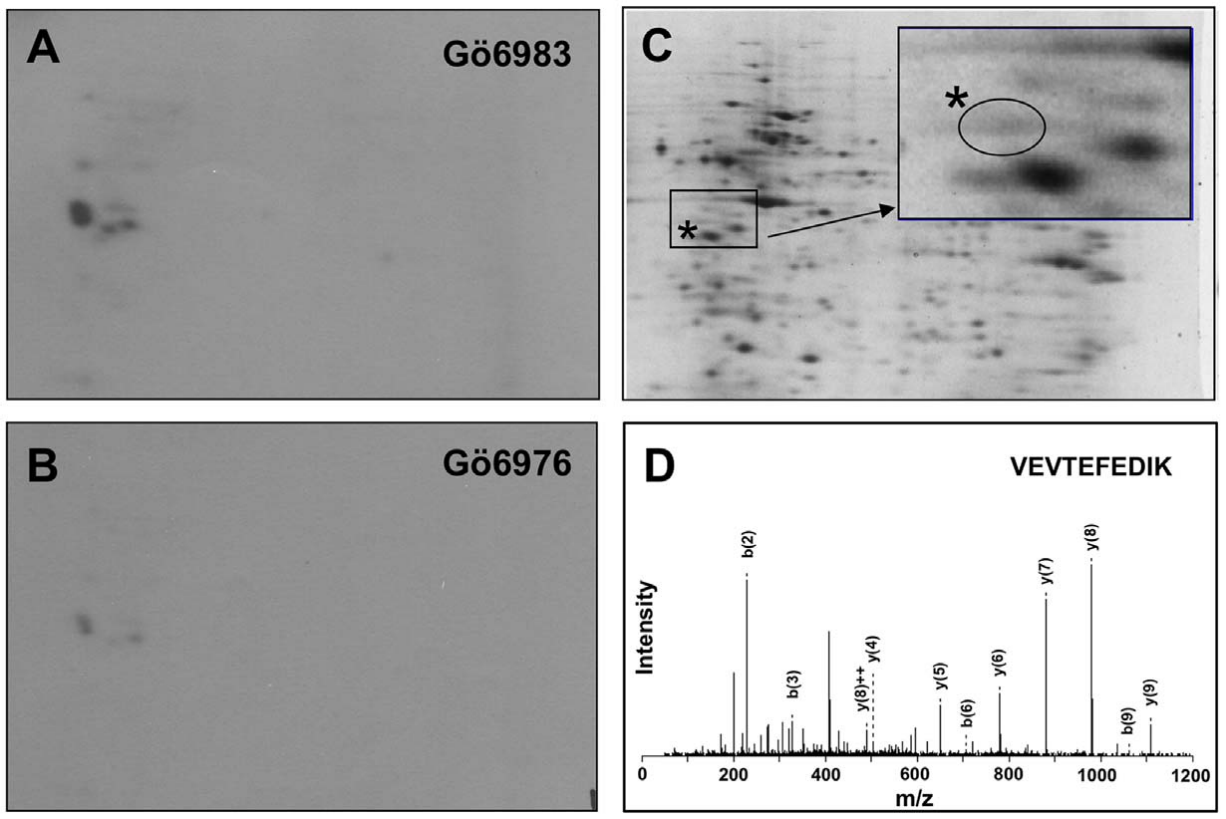
SET protein in Jurkat nuclear extract is phosphorylated by PKD2. Activated PKD2 was immunoprecipitated from lysate of Jurkat cells stimulated with anti-CD3ε antibody. The heat-inactivated (50°C, 5 min) Jurkat nuclear extract was used as a substrate for PKD2 and analyzed by 2D-gel electrophoresis. (A) Activated PKD2 was incubated with the nuclear extracts in the presence of [γ-^32^P]-ATP and Gö6983 (an irrelevant PKC inhibitor) and the radioactivity of 2D-electrophregram was detected. (B) Activated PKD2 was incubated with the nuclear extracts in the presence of [γ-^32^P]-ATP and Gö6976 (PKD2 inhibitor) and the radioactivity of 2D-electrophoregram was detected. (C) The proteins in the Jurkat nuclear extract treated with non-radioactive ATP and PKD2 were stained with syproruby. Inset; the enlarged view of the boxed area. A protein spot indicated by the asterisks, which overlaps to the radioactive spots indicated in A or in B was analyzed by mass spectrometry. (D) One of the MS/MS spectra assigned to be SET protein is shown (m/z = 604, ^110^VEVTEFEDIK^119^).

Since activated PKD2 translocates into the nucleus [12], we performed *in vitro* kinase assay using Jurkat cell nuclear extract to screen potential PKD2 substrates. The Jurkat cell nuclear extracts were equally divided into three tubes and each was subjected to the kinase reaction. Two of them were subjected to the kinase reaction with ^32^P-ATP and a pan-PKC inhibitor Gö6983 (Fig. 1A) that does not inhibit PKD kinase activity [23], or a PKD2 inhibitor Gö6976 (Fig. 1B). The third tube was subjected to the reaction with cold ATP without inhibitors (Fig. 1C). Incorporation of radioactivity was not observed in the gel when the nuclear extracts were treated with ^32^P-ATP without adding PKD2 (data not shown), showing the back ground kinase activity in the nuclear extract was well inactivated by mild heat inactivation. As shown in Fig. 1A, some radioactive spots were observed in the 2D-gel that were phosphorylated in the presence of Gö6983, but markedly suppressed in the presence of Gö6976 (Fig. 1B), indicating that the proteins were specifically phosphorylated by PKD2. The gels were stained with syproruby and the protein spots corresponding to the radioactivity were excised from the non-radioactive gel (Fig. 1C). The gel pieces were subjected to in-gel trypsin digestion and the tryptic fragments were analyzed by an ESI-Q-TOF mass-spectrometer (Qstar Pulser *i*, Applied Biosystems, Foster City, CA). The MS/MS analysis (Fig. 1D) suggested the presence of SET protein (MW = 32kd, pI = 4.1) in a faint protein spot (Fig. 1C, indicated by asterisks) overlapping to the specifically incorporated ^32^P radioactivity. The detection of SET as a potential PKD2 substrate was in accordance with our previous observation where the SET protein was enriched in the bound fraction of PhosphoProtein Purification Column (Qiagen) applied with lysate of Jurkat cells over-expressing constitutively active PKD2 [12]. These observations supported the idea that SET protein was phosphorylated by activated PKD2 in Jurkat cells upon TCR stimulation.

### PKD2 is involved in activation-induced up-regulation of SET phosphorylation in Jurkat cells

**Figure 2:**
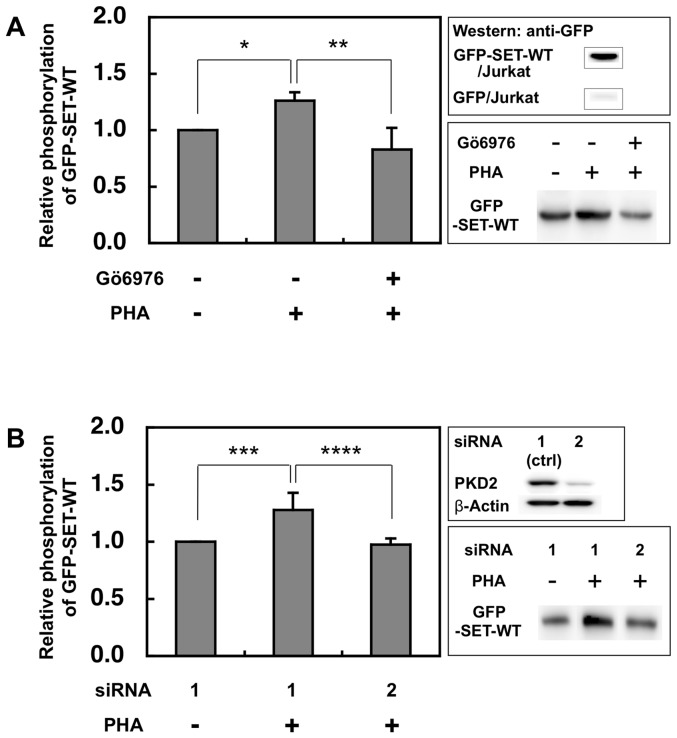
PKD2 is involved in the up-regulation of SET phoshorylation in activated Jurkat cells. (A) After Jurkat cells expressing GFP-SET were stimulated by PHA (2 µg/ml) with or without Gö6976 (3 µM), the phosphorylated GFP-tagged SET was collected by PhosphoProtein Purification kit and separated by SDS-PAGE. The phosphoprotein was transferred onto the PVDF membrane and quantified by ECL using anti-GFP antibody and the secondary antibody. The typical blot was shown in the lower-right panel. The luminescence of samples from cells without both stimulation and the inhibitor was assigned to be 1. The results were expressed as mean +SD for three independent experiments. *, p<0.01; **, p<0.05. As shown in the upper-right panel, phosphorylated GFP was not detected from the eluate of the PHA stimulated Jurkat cells expressing GFP only. (B) Jurkat cells expressing GFP-SET treated with control (ctrl) siRNA1 had no effect on the PKD2 expression, while siRNA2 markedly reduced the PKD2 expression (upper-right panel). The typical blot was shown in the lower-right panel. The luminescence of the samples from non-stimulated and siRNA1-treated cells was assigned to be 1. The results were expressed as mean +SD for three independent experiments. ***, p<0.05; ****, p<0.05, as determined by Student's *t* test.

To monitor the amount of phosphorylated SET *in vivo*, Jurkat cells expressing GFP-SET-WT were stimulated with PHA and phospho-GFP-SET-WT (GFP-pSET-WT) was recovered and quantified by western blot using anti-GFP antibody as described in the Method section. The amount of GFP-pSET-WT was up-regulated after stimulation (Fig. 2), however, the up-regulation was compromised in the presence of PKD2 inhibitor Gö6976 (Fig. 2A). Knockdown of PKD2 by siRNA also reduced the up-regulation of GFP-pSET-WT induced by PHA stimulation (Fig. 2B). GFP only expressed in Jurkat cells was not phosphorylated by the PHA stimulation since GFP band was not detected (Fig. 2A upper right panel). These data suggest that PKD2 is involved in the phosphorylation of SET in the stimulated Jurkat cells.

### PKD2 phosphorylates Ser171 of SET protein

**Figure 3:**
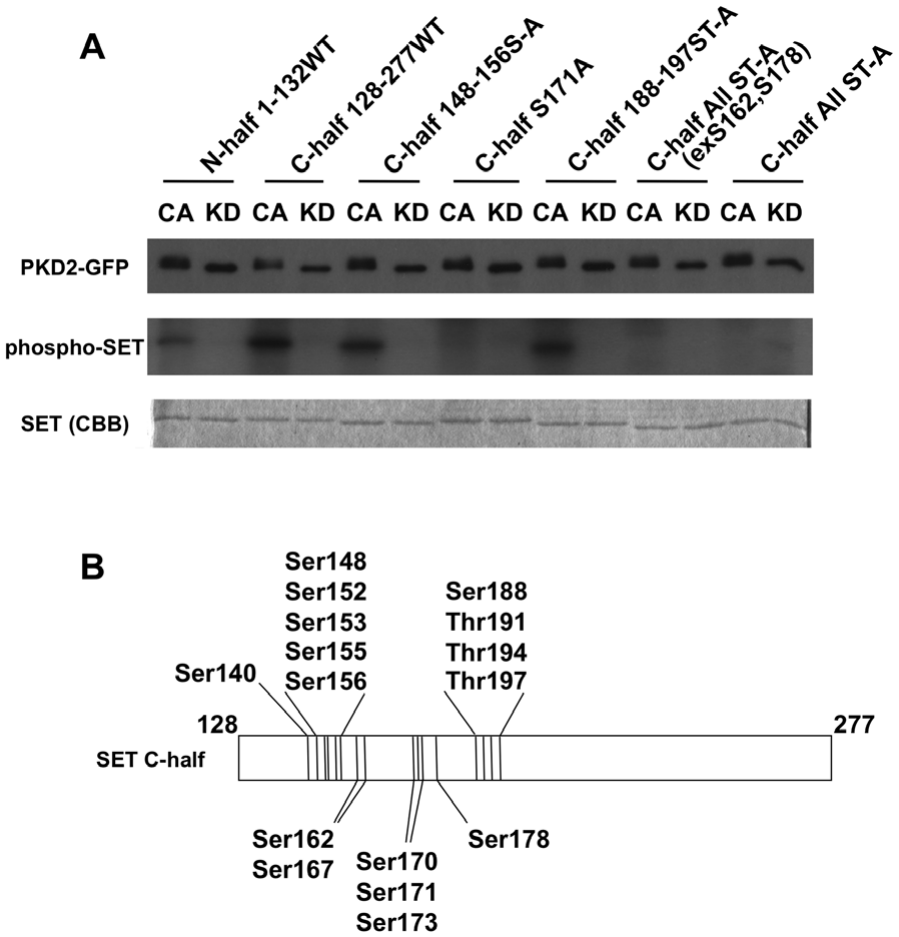
Ser171 of SET is phosphorylated by PKD2. (A) The indicated 6×His-tag SET mutant proteins were treated with immunoprecipitated constitutively active-PKD2-GFP (CA) or kinase dead-PKD2-GFP (KD) in the presence of [γ-^32^P]-ATP. The kinase assay mixture was analyzed with SDS-PAGE. Immunoprecipitated PKD2-GFP was blotted with anti-GFP antibody (upper panel) to monitor equal loading of the PKD2 mutants. Phosphorylation of the SET mutants was monitored by incorporated radioactivity (middle panel). Equal loading of each SET mutant protein was confirmed by staining with Coomassie Brilliant Blue (CBB, lower panel). The data shown are representative results from three independent experiments with similar results. (B) A diagram of Ser/Thr residues present in C-terminal half (128–277) of SET protein.

To identify the Ser/Thr residues of SET phosphorylated by PKD2, we produced a series of recombinant 6×His-tagged SET proteins with various Ser/Thr to Ala substitutions and they were subjected to *in vitro* PKD2 kinase assay (Fig. 3). Phosphorylation of the C-terminal half portion (amino acid (aa) residues 128–277) of SET protein by constitutively-active (CA) PKD2 was higher than that of the N-terminal half portion (aa residues 1–132) (Fig. 3A, CA of N-half 1-132WT and C-half 128-277WT). Therefore, we focused our investigation on the C-terminal portion of SET. In this portion, there are 16 Ser/Thr residues (Fig. 3B). Introduction of Ser/Thr to Ala substitutions in aa residues148–156 region (Ser148, Ser152, Ser153, Ser155, Thr156 to Ala) and in 188–197 region (Ser188, Thr191, Thr194, Ser197 to Ala) did not affect the incorporation of ^32^P radioactivity (Fig. 3A, CA C-half 148-156S-A and C-half 188-197ST-A). On the other hand, all Ser/Thr except for Ser162 and Ser178 to Ala substitutions abolished incorporation of ^32^P radioactivity (Fig. 3A, CA C-half All ST-A (exS162, S178)). These data suggested that the possible phosphorylation sites were either Ser140, Thr167, Ser170, Ser171 or Thr173 in the C-terminal half portion. Among these Ser/Thr residues, Ser171 fitted to the putative PKD1 phosphorylation motif (LxK/RxxS) [24], [25], [26] and Ser171 to Ala substitution abrogated the phosphorylation as well as the all Ser/Thr to Ala mutant did (Fig. 3A, CA C-half S171A and C-half All ST-A). Therefore, it turned out that Ser171 of SET protein was phosphorylated by active PKD2 *in vitro*. Since all ST-A mutant protein did not incorporate the radioactivity, the Ser/Thr residues present in the vector-derived amino acid residues were not likely to be phosphorylated by PKD2.

**Figure 4:**
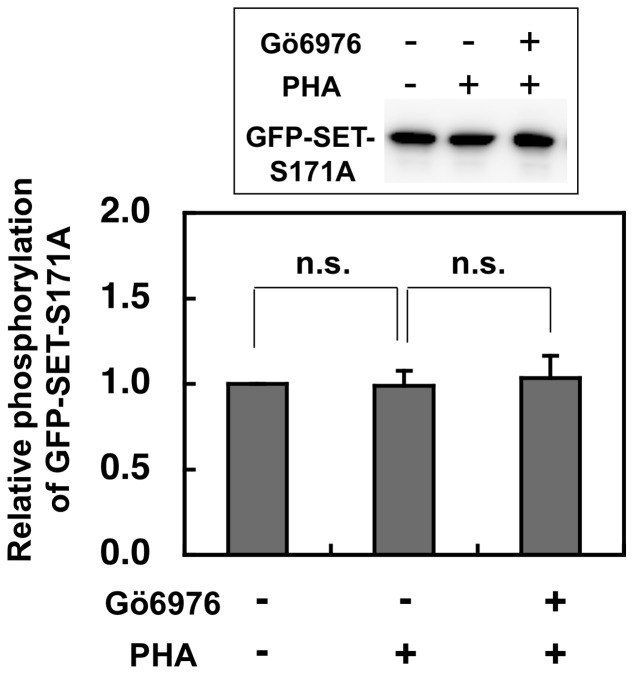
Phospho-GFP-SET-S171A levels in Jurkat were unchanged by PHA stimulation with or without PKD2 inhibitor. The Jurkat cells expressing GFP-SET-S171A were stimulated and phosphorylation status of GFP-SET-S171A was investigated by the same method as described in Fig. 2. The results were indicated as mean +SD for three independent experiments. There was no significant up- or down-regulation of recovered phospho-GFP-SET-S171A by PHA stimulation with or without PKD2 inhibitor, suggesting that Ser171 of SET is the major target site of TCR-activated PKD2.

To examine whether Ser171 is involved in SET phosphorylation *in vivo*, Jurkat cells expressing GFP-tagged SET with Ser171 to Ala mutant (GFP-SET-S171A) were stimulated with PHA in the absence or presence of PKD2 inhibitor Gö6976 and the amount of phospho-GFP-SET-S171A recovered from each cell lysate was compared by the western blotting using anti-GFP antibody. Contrary to the data shown in Fig. 2, the amount of phospho-GFP-SET-S171A was unchanged by the stimulation in the absence or presence of PKD2 inhibitor, suggesting that SET Ser171 is the major phosphorylation site of PKD2 activated by TCR stimulation (Fig. 4).

### Substitution of Ser171 to Glu in SET compromised its inhibitory effect on PP2A phosphatase activity

**Figure 5:**
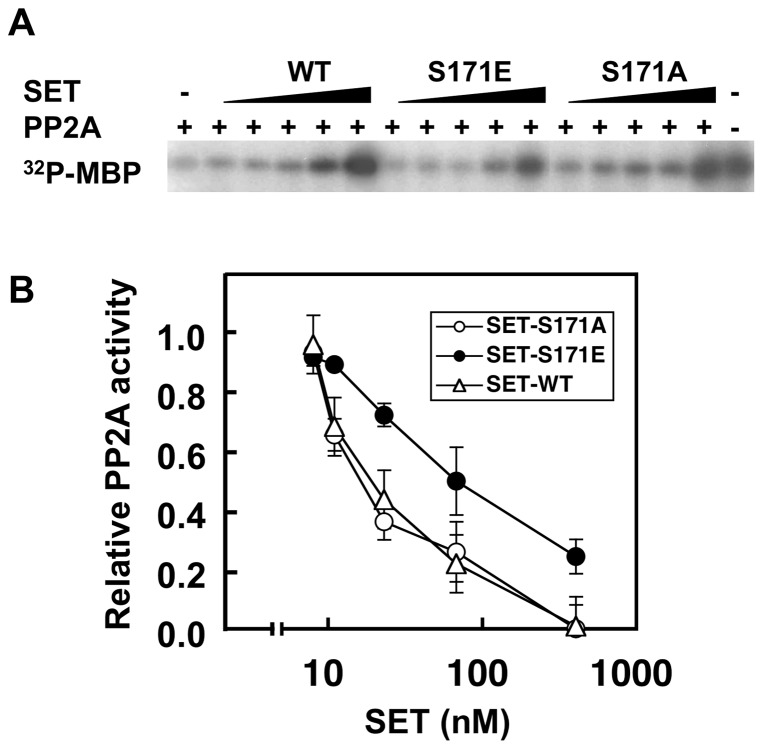
Substitution of Ser171 to Glu (SET-S171E) in SET compromised its inhibitory effect on PP2A. Substitution of Ser171 to Glu (SET-S171E), which mimics phosphorylated SET at Ser171 residue, reduced its inhibitory effect on PP2A compared with Ser171 to Ala substitution (SET-S171A) or wild-type SET did. (A) The phosphatase activity of PP2A (37 µU) under the presence of indicated concentration of recombinant SET was monitored by using ^32^P-labeled MBP as a substrate. After SET proteins and PP2A were preincubated for 15 min at 4°C, ^32^P-MBP was added and incubated for 10 min at 37°C. The reaction mixture was separated with SDS-PAGE. The inhibitory effect of SET proteins on PP2A activity was visualized by exposing the SDS-PAGE gel with PP2A-treated ^32^P-MBP to X-ray film. The data shown are representative results from three independent experiments with similar results. (B) The relative PP2A activity to the radioactivity of ^32^P-MBP without SET (assigned to be 1) was calculated from the radioactivity of MBP bands in the dried gel shown in (A). Values represent the means ±SD from three independent experiments.

Since SET protein is known to be a natural inhibitor for PP2A [2], we next tried to ask the effect of Ser171 phosphorylation on its inhibitory activity for PP2A. For this purpose, we prepared recombinant wild-type SET (SET-WT), phosphorylation-mimic Ser171 to Glu (S171E) and non-phosphorylation-mimic Ser171 to Ala (S171A) mutants. Equal amount of each purified recombinant SET protein was added to the PP2A phosphatase reaction mixture where PKA-labeled ^32^P-MBP was used as a substrate. As shown in Fig. 5, the phosphatase activity of PP2A was dose-dependently inhibited by the addition of the SET proteins. However, we noticed that the phosphorylation-mimic S171E mutant was less inhibitory than S171A or SET-WT. Even at the highest concentration (400 nM) of SET proteins tested, PP2A retained its phosphatase activity in samples with SET-S171E while that in samples with SET-S171A or SET-WT was completely abolished (Fig. 5).

### Suppression of PKD2 activity retains higher phosphorylation status of Tyr307 of PP2A

**Figure 6:**
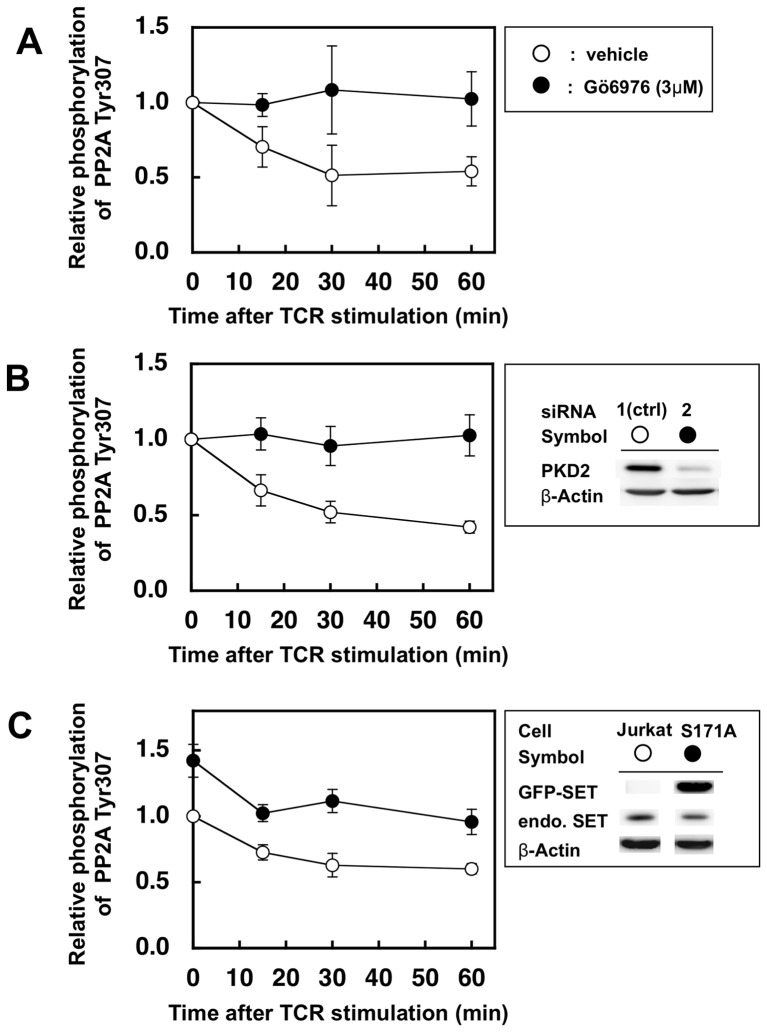
Suppression of PKD2 up-regulates Tyr307 phosphorylation of PP2A catalytic subunit after TCR stimulation. (A) Human CD4^+^ T cell clone was stimulated by L cells expressing its cognate ligands in the presence (closed circles) or absence (open circlse) of PKD2 inhibitor Gö6976 (3 µM). (B) Jurkat cells treated with siRNA were stimulated by anti-CD3ε antibody and anti-mouse IgG antibody. Treatment with siRNA2 (closed circles) significantly reduced the PKD2 expression, while siRNA1 (open circles) had almost no effect and hence used as negative controls (right panel). (C) Normal Jurkat cells (open circles) and Jurkat cells expressing GFP-SET-S171A (closed circles) were stimulated by anti-CD3ε antibody and anti-mouse IgG antibody. The latter cells expressed 4.5-fold higher GFP-SET-S171A than the endogenous SET as judged by the intensity of the bands using anti-SET antibody on the Western blot (upper box). Cell lysate were subjected to SDS-PAGE followed by western blot using anti-PP2A phospho-Tyr307 antibody and horseradish peroxidase-conjugated second antibody. The amount of each band was quantified by using ECL and LAS-4000. The same membrane was stripped and reprobed with the anti-PP2A C-subunit antibody to monitor relative abundance of total PP2A protein. Values represent the means ±SD from three independent experiments.

The data presented above suggested the possibility that PKD2 may regulate PP2A activity through SET phosphorylation *in vivo*. To ask this possibility, the relationship between PKD2 and activity of PP2A in *ex vivo* system was investigated using a TCR stimulated human CD4^+^ T cell clone or Jurkat cells. Since it is reported that phosphorylation status of Tyr307 of PP2A catalytic subunit inversely correlates with the activity of PP2A [22], we monitored phospho-Tyr307 of PP2A from stimulated T cells or Jurkat cells (Fig. 6).

The human CD4^+^ T cell clone was stimulated by co-culturing with mouse L cells expressing its cognate TCR-ligand, HLA-DR4 covalently-linked with the antigenic peptide [19], under the presence or absence of PKD2 inhibitor Gö6976 (3 µM). Jurkat cells of which PKD2 expression was knocked down by RNAi were stimulated with anti-CD3ε antibody plus the second antibody. The phosphorylation status of Tyr307 of PP2A was reduced in a time-dependent manner after TCR stimulation in both human CD4^+^ T cell clone without inhibitor (Fig. 6A, open symbols) and Jurkat cells treated with control siRNA (Fig. 6B, open symbols), suggesting that the PP2A phosphatase activity was up-regulated after TCR stimulation. On the other hand, the human CD4^+^ T cell clone treated with the PKD2 inhibitor (Fig. 6A, closed symbols) and PKD2-knocked down Jurkat cells by the siRNA (Fig. 6B, closed symbols) retained higher phosphorylation levels of PP2A compared with those of the control cells since PKD2 up-regulates PP2A activity after TCR stimulation possibly through SET phosphorylation. In accordance with this notion, stimulated Jurkat cells overexpressing GFP-SET-S171A (Fig. 6C, western blot in the upper box and closed circles) showed higher phosphorylation status of PP2A Tyr307 than that of the control Jurkat cells (Fig. 6C, open circles) at all the time points analyzed.

### Overexpression of GFP-SET-S171A down-regulates ERK activity

**Figure 7:**
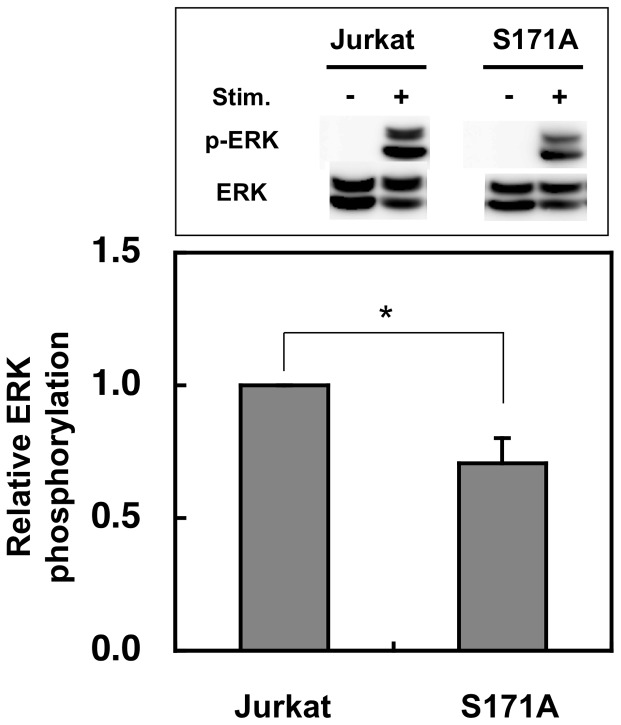
Overexpression of GFP-SET-S171A reduced the ERK activation after stimulation. Normal and GFP-SET-S171A-overexpressing Jurkat cells were stimulated by anti-CD3e antibody and anti-mouse IgG antibody for 7 min were lyzed and subjected to SDS-PAGE followed by the western blot analyses. The phospho-ERK (p-ERK) was first detected and quantified and after stripping off the antibodies, ERK1 was detected and quantified to monitor p-ERK/ERK1 ratio. The p-ERK/ERK1 ratio of Jurkat cell was assigned to be 1. The results were indicated as mean +SD for three independent experiments. *, p<0.01, as determined by Student's *t* test.

Chen et al. reported that knockdown of endogenous PKD2 down-regulated ERK activity in cancer cells [27]. It has been also reported that PP2A dephosphorylates the inhibitory phosphate at Ser259 of Raf-1, and thus PP2A activates Raf-1 and then activates ERK-dependent signaling pathway [28], [29], [30], [31], [32]. To ask whether SET is involved in the ERK phosphorylation, we compared ERK phosphorylation in Jurkat cells overexpressing GFP-SET-S171A (Fig. 6C, upper box) with that in normal Jurkat cells after stimulation (Fig. 7). Jurkat cells overexpressing GFP-SET-S171A showed slightly but significantly lower phospho-ERK/ERK ratio compared with that of the Jurkat cells, suggesting that overexpression of non-phosphorylation-mimic SET compromised PP2A activity (Fig. 6C), which resulted in the down-regulation of ERK activation in the stimulated Jurkat cells.

## Discussion

The phosphoprotein SET has been shown to have various functions in cellular activities [1], [2], [3], [33], [27], [34]. However, the relation between the phosphorylation status of SET and its functions has still been investigated. In this work, we mapped one of the phosphorylation sites for PKD2 at Ser171 of SET. Substitution of Ser171 to Glu resulted in reduction of its inhibitory effect on phosphatase activity of PP2A and overexpression of non-phosphorylation-mimic S171A SET resulted in reduction of PP2A activity.

Since the all Ser/Thr to Ala mutant of C-terminal half of 6×His-tag SET protein was not phosphorylated by PKD2, Ser/Thr residues present in the linker portion between 6×His-tag and SET were not target phosohorylation sites for PKD2. This suggested that there must be other target phosohorylation sites of PKD2 in the N-terminal half of SET protein since it also incorporated the ^32^P radioactivity when treated with active PKD2 and the sites remain to be mapped. However, since the phosphorylation of N-terminal half was much weaker than that of the C-terminal half portion, the PKD2 phosphorylation sites in the N-terminal half portion do not seem to be as effectively phosphorylated by PKD2 as Ser171. In addition, the amino acid sequences preceding Ser171 (LTKRSS) fits to the phosphorylation motif of PKD1 (LxR/KxxS) [24], [25], [26] and the LTKRSS sequence is conserved among the SET proteins of various species such as calf, chicken, mouse, xenopus and human, suggesting the functional importance of this amino acid sequence. Furthermore, Ser171 is positioned in the bottom surface of the predicted earmuff structure of SET [34]. This is in accordance with the accessibility of the kinase for phosphorylation and would provide the interaction site with other molecules such as PP2A.

The interaction between SET and PKD2 was shown in Jurkat cells since the anti-PKD2 antibody co-immunoprecipitated SET from the Jurkat cell lysate (Supporting Information S1). However, in the reciprocal experiment, the anti-SET antibody did not co-immunoprecipitate PKD2. One possibility for this discrepancy may be due to the overlapping binding site on SET for the anti-SET antibody and PKD2. Supporting this, the anti-GFP antibody co-immunoprecipitated PKD2 from GFP-SET-WT-expressing Jurkat cells together with GFP-SET (Supporting Information S1). Although we tried to show the SET phosphorylation by PKD2 in primary human T cells by *in vivo* ^32^P –labeling of SET, *ex vivo* detection of phosphorylated SET by anti-phospho-Ser antibody or by the Phos-tag detection kit (FMS Laboratory, Hiroshima, Japan), the clear difference of the phosphorylation status of SET before and after stimulation was not observed at this moment.

SET is a multi-functional protein and has an acidic domain rich in Glu and Asp residues at its very C-terminus (amino acid positions 224–277). The acidic domain is indispensable for its inhibitory effect on the histone acetyltransferases [1] and on the nucleosome assembly protein activity [35], however, the experiment using truncated mutants of SET showed that the inhibitory activity of SET toward PP2A resides in the N-terminal amino acid region of 26–119 [36]. Therefore, phosphorylation of Ser171 by PKD2 seems to regultae the inhibitory activity from outside of the N-terminal regions of SET. Recently, Vera et al. reported that casein kinase 2 (CK2) was one of the SET binding proteins and phosphorylated SET *in vitro* [37]. Although the phosphorylation sites of SET by CK2 were not identified, they speculated that phosphorylation of SET by CK2 might regulate its proteolytic degradation and cytosol-nuclear shuttling since CK2 phosphorylation of necleosome assembly protein-1, a structurally and functionally similar protein to SET, was reported to have such functions as SET did [38]. Collectively, phosphorylation of SET by kinases may regulate some of the SET functions.

The *in vitro* phosphatase assay using immunoprecipitated PP2A from activated T cells by colorimetric assays using *p*-nitrophenol or Malachite Green [39], or by the MBP-based radioactive assay were fluctuated and thus not reliable. Therefore, we adopted to monitor phosphorylation status of Tyr307 of PP2A, which inversely correlate with the phosphatase activity [22]. This method was also adopted by Neviani et al. and they reported that phosphorylation of tyrosine 307 of PP2A was increased in Bcr-Abl^+^ cells by over-expressing SET protein [40]. Without PKD2-specific inhibitor or reduction of PKD2 by RNAi, the amount of PP2A phospho-Tyr307 was decreased in time-dependent manner, suggesting the increase of PP2A activity after TCR-stimulation. Actually, phosphatase activity of immunoprecipitated PP2A from anti-CD3ε antibody stimulated Jurkat cells was up-regulated compared to that of non-stimulated cells (Supporting Information S2). This reduction of phospho-Tyr307 of PP2A was compromised by the suppression of PKD2 activity by either its specific inhibitor or RNAi, showing a relation between the PKD2 activity and some PP2A status, possibly through the phosphorylation of the natural PP2A inhibitor SET. Further such investigations using anti-phospho-Ser171 of SET should be necessary to confirm the hypothesis in the near future.

There are many reports providing the evidences that PP2A dephosphorylates the inhibitory phosphate at Ser259 of Raf-1, and thus PP2A activates Raf-1 and then activates ERK-dependent signaling pathway [28], [29], [30], [31], [32] and knocking down of PKD2 resulted in the decrease of the ERK activation in LNCaP prostate cancer cells [27]. Although we have no direct evidence at this time concerning the relation among SET phosphorylation by PKD2, up-regulation of PP2A and ERK activities *in vivo*, overexpression of non-phosohorylation-mimic mutant of SET in TCR-stimulated Jurkat cells resulted in the down-regulation of ERK phosphorylation as well as PP2A activity, suggesting the involvement of phosphorylation status of SET and PP2A activity in the ERK activation. Together, it is tempting to hypothesize that PKD2 is a positive regulator of T cell activation through SET phosphorylation and up-regulation of PP2A activity, which results in the activation of MAPK pathway. This notion is in accordant with our first finding that partially-agonistic TCR ligands induced ZAP-70 independent T cell proliferation and interferon-γ production, which was completely suppressed by PKD2 inhibitor [9], since PKD2 is activated by the over-expressed partially agonistic TCR ligands and induces T cell activation through the “SET phosphorylation-PP2A activation-ERK activation” pathway, which skips ZAP-70 activation.

In conclusion, the roles of PKD2 and its substrate SET in T cell activation were investigated and we found that PKD2 phosphorylates Ser171 of SET, which resulted in the reduction of its inhibitory effect on PP2A phosphatase activity.

## Supporting Information

Supporting Information S1 **Co-immunoprecipitation of SET by anti-PKD2 antibody from Jurkat cells lysate.** Jurkat cells (1×10^7^) and GFP-SET-WT expressing Jurkat cells (1×10^7^) were stimulated with PMA (20 ng/ml) and ionomycin (2 µg/ml) for 30 min at 37°C and lysed. Each cell lysate was immunoprecipitated with anti-PKD2, anti-SET or anti-GFP antibodies and Protein A beads. The anti-PKD2 antibody co-immunoprecipitated SET (left pannels) from Jurkat cell lysate. Reciprocally, the anti-SET antibody failed to co-immunoprecipitate PKD2 from Jurkat cell lysate (middle panels), while the anti-GFP antibody could co-immunoprecipitate PKD2 (right pannels) with GFP-SET.
(TIF)

Supporting Information S2 **PP2A phosphatase activity was up-regulated after TCR stimulation in Jurkat cells.** Jurkat cells (1×10^6^) were left untreated or stimurated with anti-CD3ε antibody and anti-mouse IgG antibody for 30 min. Each cell lysate was immunoprecipitated with anti-PP2A C-subunit antibody (anti-PP2A) or non-specific control antibody (IgG) and Protein A beads. The immunocomplex were incubated with ^32^P-MBP as a substrate. The reaction mixture was separated with SDS-PAGE. The autoradiogram was shown in the upper panel. The phosphatase activity was measured as a remaining radioactivity of ^32^P-MBP. The radioactivity of ^32^P-MBP treated with the immune complex precipitated with non-specific control antibody was assigned to be 1 (lower panel). Student's *t* test analysis for the PP2A activity of stimulated versus non-stimulated was done. *, *p*<0.01 (n = 4).
(TIF)
